# Supplementary material for: Myeloablation-associated deletion of ORF4 in a human coronavirus 229E infection
Source: NPJ Genom Med. 2017 Oct 9;2:30. doi: 10.1038/s41525-017-0033-4 (PMC5677986; doi:10.1038/s41525-017-0033-4)
Supplement: Supplementary file 1 — Figure S1 [file 41525_2017_33_MOESM1_ESM.pdf]

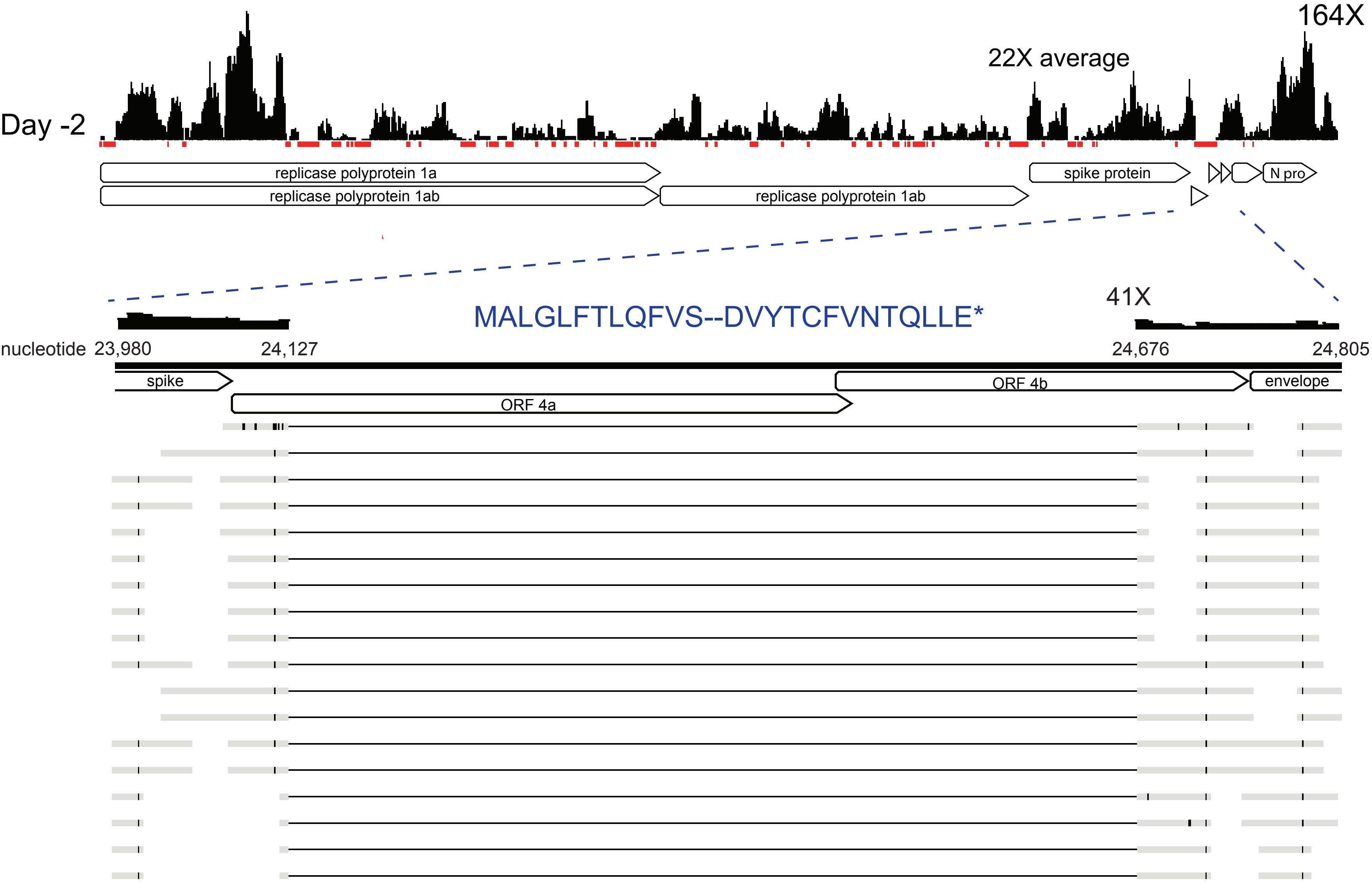

Figure S1 – Deletion detected in Day -2 specimen. mNGS sequencing coverage is shown for Day -2 specimen. This specimen was collected on the last of three days of total body irradiation. The viral strain showed numerous areas of no coverage (highlighted in red) and PCR duplicates that could be consistent with low viral titer and PCR jackpotting or RNA degradation. 18 junctional reads that spanned the deletion in the Day 15 specimen were detected.
