# Supplementary material for: Myeloablation-associated deletion of ORF4 in a human coronavirus 229E infection
Source: NPJ Genom Med. 2017 Oct 9;2:30. doi: 10.1038/s41525-017-0033-4 (PMC5677986; doi:10.1038/s41525-017-0033-4)
Supplement: Supplementary file 2 — Figure S2 [file 41525_2017_33_MOESM2_ESM.pdf]

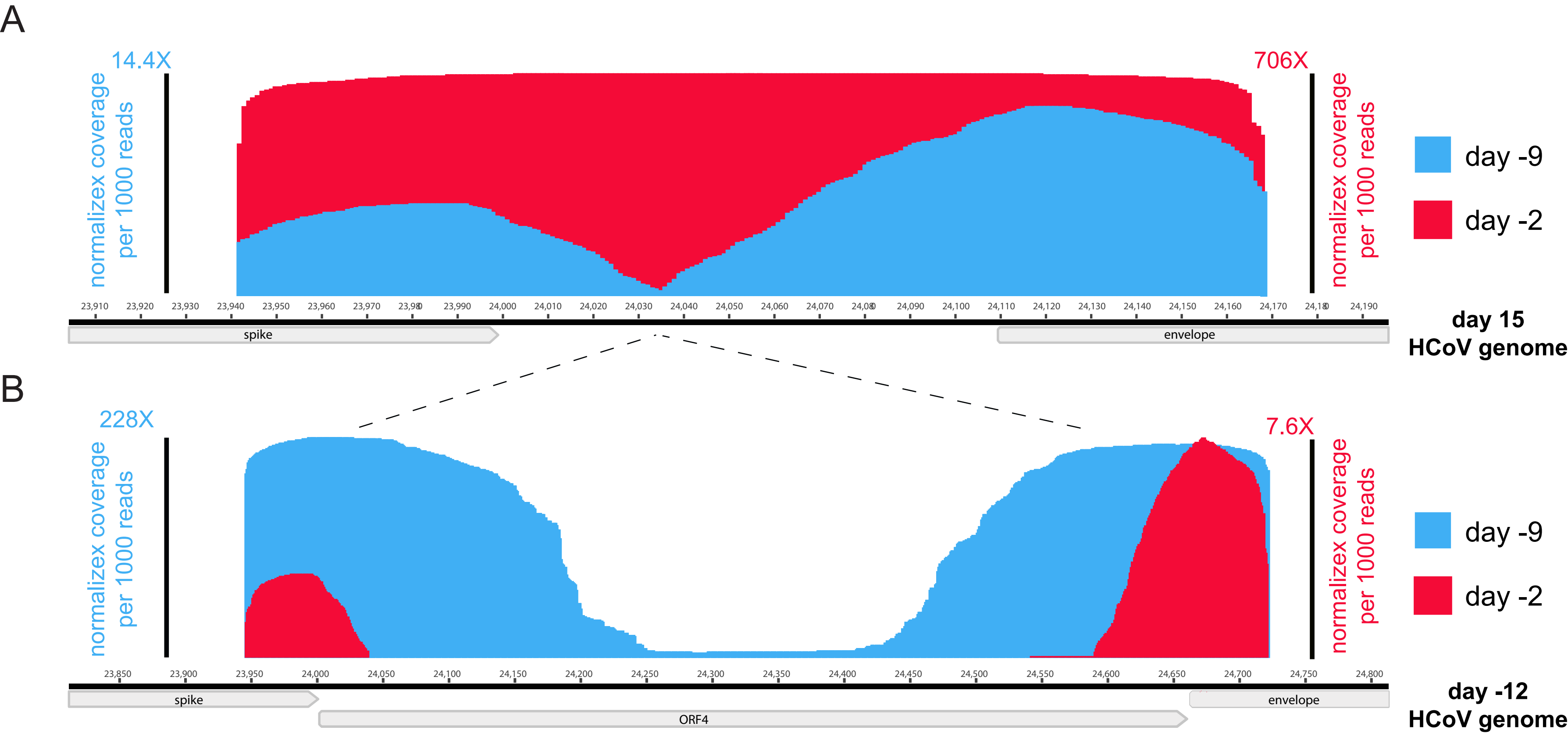

Figure S2 - Ultra deep sequencing of the day -9 and day -2 ORF4 locus after RT-PCR amplification
The ORF4 gene was amplified as in Figure 1C and sequencing adapters were ligated onto the amplicons for ultra deep sequencing. Reads from each of the day -9 and day -2 amplicons were mapped to each of the day -12 and day 15 HCoV 229E consensus genomes with no gaps and no mismatches allowed to reduce false positives. No reads from the day -9 amplicon mapped across the deletion junction from the day 15 HCoV 229E consensus genome (A), despite >11,000X depth at the locus when mapped to the day -12 HCoV 229E consensus genome (B). Only one paired-end read from the day -2 amplicon mapped to deleted portion of ORF4 present in the day -12 genome, despite coverage of >150,000X when mapped to the day 15 HCoV genome. Of note, the apparent dip in the coverage across ORF4 in the day -9 amplicon when mapped to the day -12 HCoV genome is due to the length of the sequencing reads (315x310bp run on an Illumina MiSeq) relative to the length of the intact ORF4 RT-PCR amplicon (>750bp).
