# Supplementary material for: Myeloablation-associated deletion of ORF4 in a human coronavirus 229E infection
Source: NPJ Genom Med. 2017 Oct 9;2:30. doi: 10.1038/s41525-017-0033-4 (PMC5677986; doi:10.1038/s41525-017-0033-4)
Supplement: Supplementary file 3 — Figure S3 [file 41525_2017_33_MOESM3_ESM.pdf]

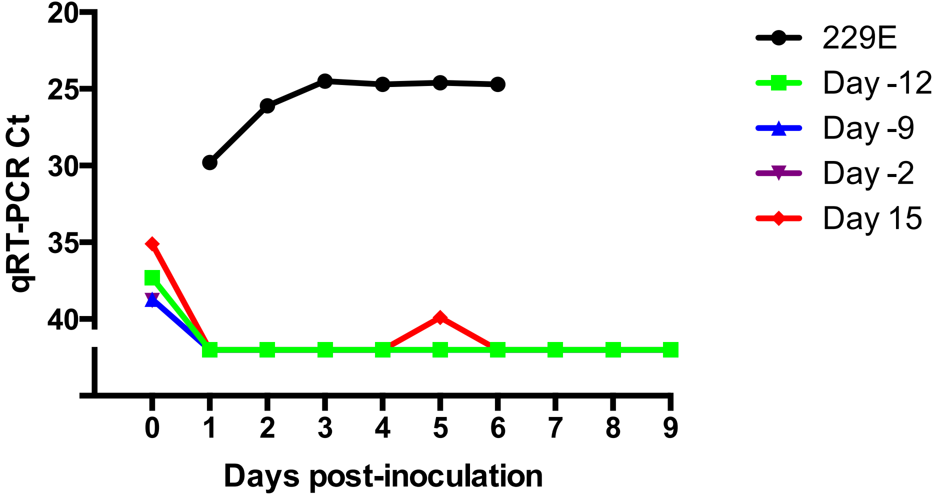

Figure S3 - The HCoV 229E strains sequenced in this study failed to grow in culture.
None of the patient's strains of HCoV 229E grew in MRC5 cell culture as assessed by cytopathic effect or qRT-PCR. The patient's nasal swabs in viral transport media were inoculated onto MRC5 culture tubes for 30 minutes and supernatants were taken every day for 9 days. The qRT-PCR cycle threshold for each supernatant is depicted along with the original inoculum Ct. A HCoV 229E ATCC strain served as a positive control.
